# Observance of the Atlantic Diet in a Healthy Population from Galicia (NW Spain): A Comparative Study Using a New Scale-Based Procedure to Assess Adherence

**DOI:** 10.3390/foods14152614

**Published:** 2025-07-25

**Authors:** Inés Rivas-Fernández, Paula Roade-Pérez, Marta López-Alonso, Víctor Pereira-Lestayo, Rafael Monte-Secades, Rosa Argüeso-Armesto, Carlos Herrero-Latorre

**Affiliations:** 1 Escola Universitaria de Enfermería, Servizo Galego de Saúde, Universidade de Santiago de Compostela, Campus Terra, 27002 Lugo, Spain; i.rivas.fernandez@usc.es; 2 Departamento de Química Analítica Nutrición y Bromatología, Facultade de Veterinaria, Universidade de Santiago de Compostela, Campus Terra, 27002 Lugo, Spain; paula.roade@usc.es; 3 Departamento de Patoloxía Animal, Facultade de Veterinaria, Universidade de Santiago de Compostela, Campus Terra, 27002 Lugo, Spain; marta.lopez.alonso@usc.es (M.L.-A.); victor.pereira@usc.es (V.P.-L.); 4 Hospital Universitario Lucus Augusti, Calle Dr. Ulises Romero 1, 27003 Lugo, Spain; rafael.monte.secades@sergas.es (R.M.-S.); rosa.argueeso.armesto@sergas.es (R.A.-A.); 5 Aquatic One Health Research Center (iARCUS), Departamento de Química Analítica Nutrición y Bromatología, Facultade de Ciencias, Universidade de Santiago de Compostela, Campus Terra, 27002 Lugo, Spain

**Keywords:** Atlantic Diet, adherence assessment, consumption patterns, Galicia (NW Spain)

## Abstract

The Atlantic Diet (AD) is based on traditional dietary patterns in Galicia (northwestern Spain) and northern Portugal and is known for its health benefits. The AD focuses on fresh, local, and seasonal foods, especially fish, seafood, vegetables, legumes, whole grains, fruit, olive oil, and a moderate consumption of wine. However, it has received less attention from researchers than other dietary patterns. The present study had two main objectives: (i) to evaluate the dietary habits of a Galician population in relation to the AD and (ii) to create a numerical index to measure adherence to the AD. In 2022, a validated food frequency questionnaire was administered to 500 healthy adults living in Galicia. The data on participants’ dietary habits showed notable deviations from the ideal AD, especially regarding consumption of fruits, grains, and seafood. However, an adequate intake of legumes and nuts was observed, along with a reduction in the consumption of processed foods (except among younger participants) relative to that revealed in previous surveys. To assess adherence to the diet, statistical and chemometric analyses were applied, leading to the development of a new index: the Atlantic Diet Scale (ADS). The ADS was compared with three existing tools and proved to be a simple, flexible, and effective method for assessing dietary adherence based on optimal intake levels across food groups. When applied to dietary data, the ADS yielded adherence levels similar to two of the three traditional methods, with some differences relative to the third. These findings highlight the need for standardized evaluation tools, including clear definitions of food groups and consistent scoring systems, to better assess and promote adherence to the Atlantic Diet.

## 1. Introduction

Dietary patterns have long been recognized as key determinants of health, influencing the prevalence of chronic conditions such as cardiovascular disease, obesity, and diabetes [1]. The Atlantic Diet (AD), also known as the Southern European Atlantic Diet, is based on traditional dietary patterns in parts of southern Europe, particularly in Northern Portugal and Northwestern Spain [2,3]. The autonomous region of Galicia (NW Spain) is renowned for its culinary heritage, featuring a wide variety of high-quality foods, including plant-based, animal-based, and marine foods, many of which are produced by sustainable agricultural and fishing practices [4] and/or carry geographical quality labels (e.g., designation of origin, protected geographical indication, and traditional specialty guaranteed) [5,6]. The AD is deeply embedded in Galician culinary traditions, emphasizing fresh, seasonal, and locally sourced ingredients [7]. It prioritizes plant-based foods (vegetables, fruits, cereals, whole-grain bread, legumes, and nuts) and regular consumption of fish and seafood, dairy products, and quality meats (beef, pork, poultry, and game). Olive oil is widely used for seasoning and cooking, and simple culinary techniques like boiling, roasting, baking, and stewing are used [8]. These principles are covered by the traditional Atlantic Diet pyramid proposed by Tojo and Leis [9].

Several findings position the AD as a health-promoting dietary pattern. Monounsaturated fatty acids from olive oil and ω-3 polyunsaturated fatty acids from fish and seafood are known to support cardiovascular health [10]. Nuts contribute antioxidant and anti-inflammatory compounds with cardioprotective properties [11]. Animal-based foods, particularly fish, are rich in protein and essential microminerals [12,13]. A positive association between adherence to the AD and higher concentrations of iron and selenium in maternal milk has been reported in a recent study [14]. Dairy consumption has been linked to improved muscle function, reduced blood pressure and LDL cholesterol, and a lower risk of diabetes and certain cancers [15]. Chemical and pharmacological evaluations, including data from the Protein Data Bank, have confirmed the anticancer potential of the AD [16]. In fact, the high life expectancy in Galicia of 83.6 years at birth in 2022 (2.1 years older than the European Union average) [17] and the low cardiovascular mortality are results that, although multifactorial, are increasingly attributed to adherence to the AD [18]. In summary, several studies have shown that the AD offers nutrients of high biological value, promotes healthy culinary practices and physical activity—as other essential factors in any healthy lifestyle—and contributes to reducing the carbon footprint [8,19,20,21]. However, recent dietary shifts in Galicia reveal a departure from this pattern, with consumption of food of lower nutritional quality and approximately 15% higher greenhouse gas emissions than the AD [22,23]. Globalization, urbanization, time constraints, aggressive food marketing, and the decline in traditional home cooking have all contributed to the increased consumption of processed and high-fat food [24]. Additionally, there is some debate regarding how best to assess adherence to the AD. The Galician health authority (Dirección General de Saúde Pública, Consellería de Sanidade, Xunta de Galicia) has defined “Optimal Atlantic Diet Consumption Values” (OADCVs) for twelve food groups [25]. These values provide a qualitative framework for assessing adherence to the AD. However, other researchers have also proposed numerical indices to assess adherence to the AD based on different qualitative and quantitative approaches [2,8].

This study had two main objectives: (i) to evaluate the eating habits of a Galician population in relation to the Atlantic Diet (AD), and (ii) to develop a numerical index to measure adherence to the AD. To achieve the first objective, dietary data were collected using a quantitative Food Frequency Questionnaire (FFQ). The data obtained (hereinafter Galician Consumption Pattern 2022, GCP-2022) were compared with the OADCVs. For the second objective, a numerical procedure for assessing adherence to the Atlantic diet was proposed: the Atlantic Diet Scale (ADS). Scores (0/1) were obtained for the different food categories in relation to a 50–150% range around the OADCV. The total adherence index was calculated as the sum of the scores for the different food categories. The results obtained using the ADS were compared with those obtained using other adherence assessment tools and criteria. With these objectives, the study aims to contribute to a more precise understanding of current dietary practices in Galicia and provide a robust framework for assessing adherence to the Atlantic Diet.

## 2. Materials and Methods

### 2.1. Study Population and Ethical Statement

The present study was conducted as the initial phase of a wider research project investigating the role of microminerals in cancer patients in the province of Lugo (NW Spain) in relation to diet, exposure, age, sex, and other factors. This article presents the results of a survey on the dietary habits and adherence to the Atlantic Diet pattern in a healthy sample population living in Galicia (NW Spain). In total, 500 participants (326 women and 174 men) aged 18 to 80 years were recruited voluntarily in 44 municipalities in the province of Lugo. The inclusion criteria required participants to be adults residing in Galicia, aged 18 to 80, of either sex, in good general health, with no known illnesses. The exclusion criteria rejected all individuals who, according to their own statements during the recruitment process, declared they had or had been diagnosed with chronic or acute diseases. Participants were given detailed information about the study objectives, use of the biological samples and information obtained, and the data management procedures. Signed informed consent was obtained from all participants, along with authorization for possible future access to their medical records, if necessary for the purposes of the study. The study participants were recruited via the Lucus Augusti University Hospital (HULA) (Lugo), where anthropometric measurements were recorded and blood samples were obtained for analysis of biochemical parameters and serum micromineral status.

Ethical approval for the study was obtained from the health authorities in Galicia by application to the Galician Ethics Committee (Comité Territorial de Ética de la Investigación de Santiago-Lugo, Consellería de Sanidade, Xunta de Galicia), with registration code 2022/034. The study was also conducted in accordance with the Declaration of Helsinki and Spanish clinical research regulations. All data collected were treated confidentially, following Spanish data protection laws.

### 2.2. Questionnaire on Dietary Habits

Information about the dietary habits of the Galician population was obtained by administration of a quantitative food frequency questionnaire (FFQ) to the 500 study participants between January 6 and December 5, 2024. Finally, after purging the survey results (by eliminating questionnaires with missing or inconsistent responses), 456 individuals were finally included in the study (details in Table S1, Supplementary Materials). The FFQ administered is the one used by the Government of Galicia [25] to carry out studies on nutrition in the Galician population, including questions related to diet: frequency of consumption and serving sizes of different food categories. It also includes some questions about aspects related to health and lifestyle, as well as information on sociodemographics, employment situation, family data, and anthropometric measures related to nutritional status. The total time to complete the survey was approximately 45 min (the whole FFQ questionnaire is available at the following link: https://www.sergas.es/cas/Publicaciones/Docs/SaludPublica/PDF-2153-es.pdf, accessed on 5 May 2025).

### 2.3. Data Matrix

In order to assess adherence to the AD and enable comparisons with previous studies, the average daily intake (grams per day) of each category of food was calculated according to the serving sizes established by Velho et al. [3] and Carbajal [26]. In the data matrix constructed, X_456×13_, the rows correspond to 456 participants, and the columns to the average daily intake (in grams per day) for the thirteen food categories considered: fruit, vegetables, legumes, grains, nuts, dairy, eggs, meat, seafood, processed foods, sweets, oils/fats, and wine. Other relevant information, such as sex, age (18–39, 40–59, and >60), and geographic area (coastal/inland), was also included in the dataset.

The Galician Consumption Pattern identified in this study (GCP-2022) was compared with the Optimal Consumption Values of the Atlantic Diet (OADCV) established by the Galician health authorities. For this comparison, all food categories, except wine, were considered.

### 2.4. Statistical Analysis

The previous X_456×13_ described data matrix was analyzed using various classical descriptive statistical methods. In addition, two different chemometric visualization techniques—Principal Component Analysis (PCA) and Hierarchical Cluster Analysis (HCA)—were used to assess relationships between different types of food categories and between these and other influencing factors. PCA is a chemometric method used to reduce data dimensionality while preserving most of the variance. It transforms original variables into principal components (linear combinations of the variables), thus enabling visualization through score plots (samples), loading plots (variables), and bi-plots (relationships) [27]. HCA is an unsupervised technique that groups samples or variables (e.g., food categories) based on similarity [28]. The Euclidean distance was used to measure similarity, and Ward’s method was applied for clustering. This approach yields compact and interpretable clusters, minimizes intra-cluster variance and, therefore, it is commonly used in dietary pattern classification for producing compact, interpretable, and well-separated clusters. The results are shown in a dendrogram representing hierarchical relationships. Both PCA and HCA used autoscaling, where each variable was standardized by subtracting the mean and dividing by the standard deviation to avoid bias from differing magnitudes. This results in standardized variables with a zero mean and variance unity [29].

The data distribution was initially assessed using visual methods (Q–Q plots and histograms) and the Shapiro–Wilk test. In an attempt to meet the assumptions of parametric analyses, several commonly used transformations (logarithmic, square root, and inverse) were applied. However, none of these transformations sufficiently improved normality or variance homogeneity. Therefore, it was decided to use non-parametric tests for the main analyses. Specifically, the Mann–Whitney U test [30] was used to assess the effect of sex within each age group, and the Kruskal–Wallis test [31] was used separately for men and women to detect any differences in relation to age groups. Additionally, to confirm the robustness of the findings, generalized linear models (GLMs), assuming a Poisson distribution, were used to model processed food consumption as a function of sex and age group. The results obtained from GLMs were consistent with those of the non-parametric tests. The potential influence of outliers was also examined, and the main conclusions remained unchanged regardless of whether extreme values were included or excluded.

ANOVA with post hoc tests (Tukey’s HSD) [32] was used to identify any significant differences between the values of the normalized adherence indices obtained by the different systems tested.

All statistical and chemometric analyses were performed using IBM SPSS for Windows v.27 (IBM Corporation, Armonk, NY, USA) and Statgraphics Centurion XIX v.19.6.05 (Statistical Graphics Corporation, Rockville, MD, USA).

### 2.5. Proposed Procedure for Assessment of Adherence to the AD: The Atlantic Diet Scale (ADS)

A scale indicating the degree of adherence to the traditional AD, called the Atlantic Diet Scale (ADS), was constructed on the basis of the scores (0/1) obtained for 13 food categories previously described. In the present case, an optimal consumption range was considered for each category based on the optimal Atlantic Diet consumption values (OADCV), provided by the health authorities of Galicia [25] and presented in Table 1. In each case, the 50–150% percentiles were calculated. For most food groups (fruit, vegetables, cereals, dairy products, meat, and fish), the 25th and 75th percentiles fall approximately within this range, reflecting the typical variability in consumption across the population. Therefore, the selected range captures the dietary habits of most individuals without penalizing minor deviations. A value of 1 was assigned to each of the categories considered when the result was in the established range 50–150% of the optimal value, and 0 otherwise. For beneficial components (vegetables, legumes, fruit, and seafood), individuals with consumption below 50% OADCV were assigned a value of 0, and those with consumption equal to or higher were assigned a value of 1. For components presumed to be detrimental (sweets and processed foods), the valuation was reversed. Daily intake of up to 1 glass of wine for women and up to two glasses for men accurately reflected the traditional meal-drinking pattern characteristic of the Atlantic diet [8,21]. For wine, the score was based on moderate consumption with a limit of a glass/day for women and 2 glasses for men. When wine consumption was lower than or equal to the limit, a score of 1 was assigned; otherwise, it was 0. Therefore, the total score in this AD scale was obtained as the sum of the partial scores for each of the thirteen categories.

Thus, the AD scale ranged from 0 (null adherence to the traditional AD) to 13 (total adherence).

### 2.6. Other Procedures for Assessing Adherence to the Atlantic Diet

The adherence data obtained using the proposed ADS procedure on 456 individuals were compared with those derived from two other adherence assessment methods developed by Oliveira et al. [2] and García-Gómez et al. [8], applied to the same group.

In the first case, Oliveira et al. developed a Southern European Atlantic Diet adherence index (hereinafter SEAD Index) to evaluate compliance with this traditional dietary pattern. The SEAD Index is based on nine key food groups, particularly focused on the consumption habits of the Northern Portuguese population: fresh fish (excluding cod), cod, red meat and pork products, dairy products, legumes and vegetables, vegetable soup, potatoes, whole-grain bread, and wine. Similar to other well-established dietary indexes, such as the Mediterranean Diet index proposed by Trichopoulou et al. [33], each food group (except for wine) was assigned a score of 1 if consumption met or exceeded the sex-specific median, while lower consumption was awarded a score of 0. One glass of wine for women and up to two glasses for men per day, as indicated above, constitutes a traditional pattern in the Atlantic Diet. This level of consumption was assigned a score of 1, whereas higher and zero consumption were both awarded a score of 0. The SEAD index (range, 0–9) was calculated as the sum of the score for the nine individual food groups, and the cumulative score reflects adherence to the Atlantic Diet. Higher scores indicate greater conformity to the diet. The procedure was applied using the FFQ data for the same 456 individuals described in Section 2.2, with a minor modification to adapt the criteria of the SEAD Index to Galician dietary habits: thus, cod was included in the fish category, and vegetable soup was included in the vegetables/legumes group. Thus, in the case at hand, the SEAD index scores ranged from 0 to 7.

In the second Atlantic Diet assessment procedure, proposed by García-Gómez et al. [6] and hereinafter referred to as GGP, adherence was judged on the basis of the information provided in a questionnaire composed of 13 items (Table 2): eleven of these were related to food and beverages, one focused on culinary techniques, and another addressed the consumption of local and seasonal products (both of which are closely linked to the Atlantic Diet pattern). These 13 items were considered in order to determine an adherence index. Each component was assigned a score of either 0 or 1 point. In this case, the final score is in the range of 0–13.

Finally, another assessment approach, the Atlantic Diet Scale Median-based procedure (ADS-MBP), was also considered. This system is similar to the ADS procedure but is modified by using the criteria by Trichopoulou et al. [33] and Oliveira et al. [2]. Thus, instead of the 50–150% range for OADCV, the score for each group was determined using the sex-specific median benchmark. For the same 13 groups described in Section 2.4, a score of 1 was assigned if consumption met or exceeded the sex-specific median value, while lower consumption was awarded a score of 0.

As the scales used in the different adherence assessment systems vary (from 0 to 13 in some cases, and from 0 to 7 in other cases), the normalized index (NI) was used to compare the results obtained with the four different procedures. The NI was obtained by dividing the index score value obtained by the maximum index size. This process provided a normalized index for all adherence procedures, with adherence values in the range 0 to 1, where 0 represents null adherence to the AD and 1 represents complete adherence.

## 3. Results and Discussion

### 3.1. Galician Consuming Pattern-2022 (GCP-2022)

#### 3.1.1. Multivariate Data Visualization

**Figure 1:**
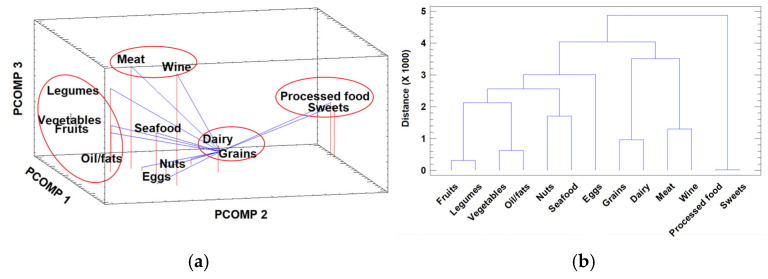
(**a**) Loading plot of the food categories in the space defined by the first three principal components. (**b**) Dendrogram of the food categories obtained by hierarchical cluster analysis (Euclidean squared distance and agglomerative Ward method).

Preliminary examination of the X_456×13_ matrix was carried out by PCA and HCA, with the objective of revealing the latent relationships between variables (food categories) and samples. The loading plot of the variables in the space defined by the first three principal components (representing 43.32% of the total variance) is shown in Figure 1a (eigenvalues and cumulative variance for the principal components are presented in Table S2, in Supplementary Materials).

Different groups of variables were identified in this factor space. A strong relationship between processed foods and sweets was detected; thus, the associated consumption of these two categories of foods indicates that individuals who consume processed foods also ingest greater amounts of sweets. The same pattern of association was found for grains and dairy products. Finally, the association between meat and wine is clear, as consumption of wine (especially red wine) is mainly linked to beef, pork, and game dishes. Another obvious group of variables (associated with adherence to the AD) is formed by fruit, vegetables, legumes, and oil/fats. Individuals who consume fruit and vegetables also consume legumes and olive oil, a fundamental, healthy fat with which salads and other vegetable dishes are seasoned. The relationship between the other three remaining categories, i.e., seafood, eggs, and nuts, is not as clear.

In order to verify the associations between categories revealed by PCA, a second visualization technique was used: HCA. The results obtained using this hierarchical associative technique are presented in the form of a dendrogram in Figure 1b. The groups of variables identified were exactly the same as before: processed food and sweets; grains and dairy; meat and wine; fruits, vegetables, and oils. The concordance between the results of both techniques, which are based on different mathematical approximations, supports the reliability of the results.

**Figure 2:**
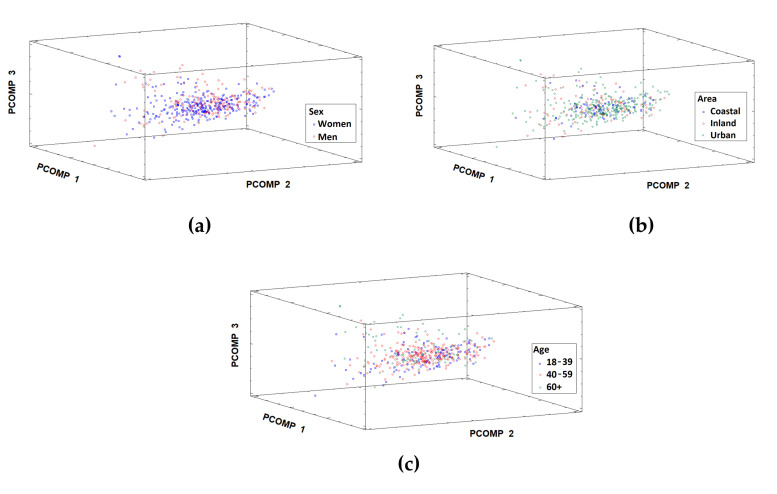
Score plot of the samples in the space defined by the first three principal components according to (**a**) sex, (**b**) geographic area, and (**c**) age group of the individuals.

PCA was also used to study possible relationships between samples (individuals), based on factors such as sex, age group, and geographical area of origin. No groupings of samples were observed for the factors considered (Figure 2a–c). This indicates the absence of any significant differences in the diet, which, overall, is conditioned by these factors. This finding was verified below using other statistical tools and procedures for assessing adherence to the AD.

#### 3.1.2. Intake Patterns: Influence of Gender, Age and Geographic Origin

The data collected by means of the FFQ regarding consumption of the different food categories indicated above by the sample population in Galicia in 2022 are presented in Table 1. The Optimal Daily Consumption Values (OADCVs) [25] are also included for comparative purposes. The intake values are very different from the OADCVs, considered ideal by the health authorities. Consumption levels were statistically significantly different from the officially optimum recommended levels (perhaps somewhat demanding), except for legumes. Perhaps the greatest difference is in regard to consumption of fruit, grains, and seafood, which is lower than recommended (36.42%, 43.62%, and 42.40% of the optimum values). There is therefore an evident need to encourage consumption of the items in these categories. On a positive note, although the average intake of processed products remains high, the level of intake has decreased (except in the younger age group) relative to that reported in the survey conducted in 2007 [25]. The shift toward a healthier diet may be due to successful nutritional interventions or access to reliable, verified information from certified nutritionists, as well as the belief that returning to traditional consumption patterns and consuming less processed food has a positive impact on health. Finally, there seems to be an extraordinary increase in the consumption of sweets, which significantly exceeds the recommended level and is another focus of action for the coming years.

**Figure 3:**
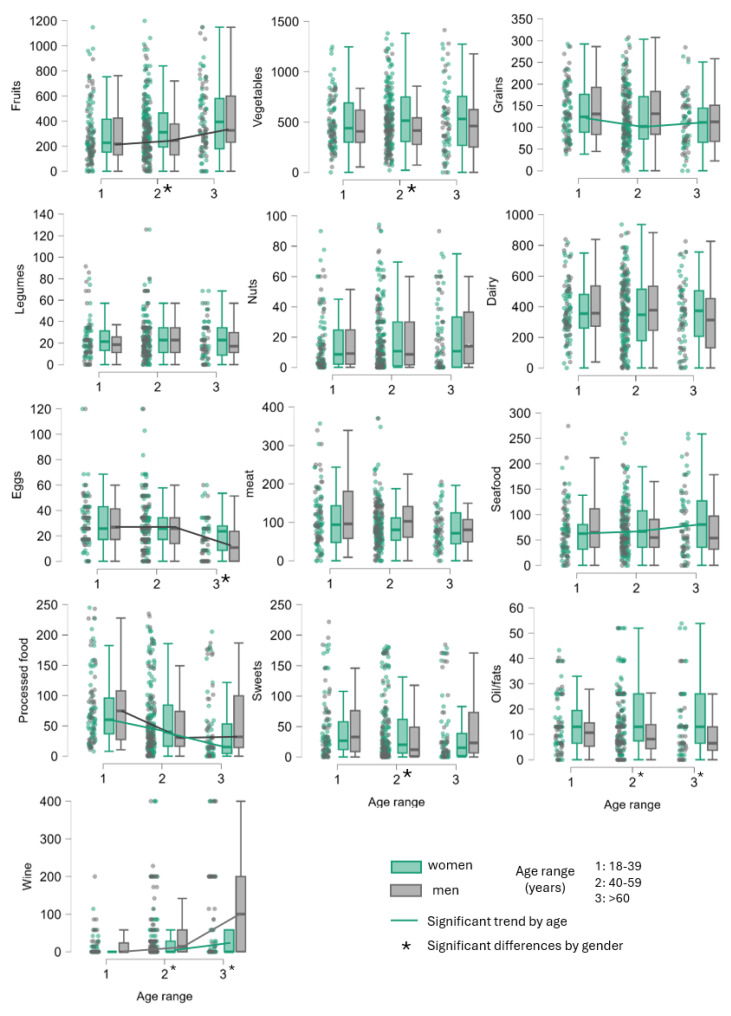
Box-and-whisker plot for the categories with statistically significant differences (*p* < 0.05) on the basis of sex, geographic area, and age groups of the individuals. Asterisk mean significantly different groups.

The dietary habits related to different food groups in men and women across various age brackets are illustrated in Figure 3. Statistical data on consumption of the different food categories in relation to sex, age, and location are detailed in Table S3 in Supplementary Materials.

As already mentioned, three age groups were established: group 1 from 18 to 39 years, group 2 from 40 to 59 years, and group 3 of 60 years and older. The most significant gender-based disparities occurred in group 2, in which women consumed significantly more fruit and vegetables and also more sweets than men. Furthermore, in age groups 2 and 3, women tended to consume more oil and less wine than their male counterparts. Regarding age-associated changes, an age-related decline in the consumption of processed foods and an increase in wine consumption were observed in both sexes. Additionally, women in the older age groups tend to consume more fish but fewer cereals, whereas the older age groups of men tend to consume more fruit and significantly fewer eggs.

The differences in dietary patterns according to sex observed in Galicia are consistent with the findings of previous studies conducted in other countries. Regarding plant-based foods, there is consensus in the scientific literature that women typically consume more fruit and vegetables than men across various dietary frameworks, including the Mediterranean diet [34], Western eating habits, as observed in the UK and Canada [35,36], and Northern European nutritional practices [37].

Wardle et al. [38] suggest that the preference for fruit and vegetables shown by women can be partly attributed to a greater focus on weight management and stronger beliefs in the benefits of eating healthily. In this study in Galicia, gender differences were particularly notable in the participants aged 40 to 59 years, at a stage of life when women increasingly prioritize their health. Conversely, our study also revealed that middle-aged women consume more sweets than men, contrasting with findings from the UK where men aged 31–59 were the predominant consumers of sugary snacks, and women in the same age group often purchased these snacks for their children or grandchildren rather than for themselves [35]. This discrepancy suggests that cultural or societal influences may be shaping these dietary choices. Regarding fats and alcohol, gender-related differences were also evident. In Galicia, women in the 40–59 age group tend to consume more oil (predominantly olive oil, consistent with the AD) and drink less wine than their male counterparts. This mirrors qualitative observations by other studies in which female participants reported that they consumed more olive oil, within a vegetable-rich diet that aids weight control [39], but consumed very moderate amounts of alcohol, primarily in social settings, unlike older men, who consume alcohol more regularly during their leisure time [35].

Both the present study and recent findings confirm that older individuals tend to retain more traditional and healthier dietary patterns than younger individuals. In the sample population in Galicia, it was observed that consumption of ultra-processed food decreased with age in both sexes, while consumption of wine increased. This is consistent with findings in other populations: Juul et al. [40] and Dicken et al. [41] reported that younger adults consumed higher levels of ultra-processed or convenience foods, whereas older adults showed a clear preference for fresh ingredients and home-cooked meals. Regarding alcohol intake, data from the WHO European Region [42] show that alcohol consumption per capita tends to increase with age, particularly among men, although the amounts remain moderate in many Mediterranean countries. These changes may reflect the amount of time available for food preparation, as well as growing health awareness and adherence to cultural practices such as drinking wine with meals, factors traditionally associated with the Mediterranean diet and more prevalent in older generations. We also identified some specific dietary changes by sex with aging. In women, advancing age was associated with an increase in fish consumption and a reduction in consumption of cereals (bread, pasta, etc.). Qualitative data reported by Chambers et al. [35] indicate that dietary preferences shift with age, with younger participants favoring pasta, and older participants consuming more fish and often replacing carbohydrates like pasta with leaner proteins for a healthier diet. In the male participants, fruit intake increased with age, and egg consumption decreased greatly with age. The reduction in egg consumption is likely influenced by a long-standing belief about the potential for eggs to increase cholesterol levels, reflecting how health concerns have become more important to dietary choices as men age [43]. It is important to note, however, that recent research indicates that eggs do not necessarily pose a significant health risk for cardiovascular health in most individuals [44], though previous perceptions may still guide the dietary choices of older generations. Regarding fruit consumption, younger men typically consume less fruit and vegetables than their female counterparts. This pattern changes as they age, with older men consuming more fruit. This shift likely reflects a growing awareness of health issues and a conscious adjustment of dietary habits in men, effectively narrowing the gap in fruit intake between older men and women [45].

Analysis of the data based on the type of area where the participants live also yielded some interesting results. Statistically significantly lower consumption of cereals (*p* = 0.014) and dairy products (*p* = 0.028) was identified in participants living in coastal regions, relative to those living in urban or inland areas, where respectively higher and equivalent consumption of these products was observed. On the other hand, significantly higher meat consumption (*p* = 0.002) was also observed among participants living in inland areas. This finding can be easily explained, as a large part of the population of inland Galicia, which is markedly rural, lives on (or near) livestock farms with extensive access to pork, as well as beef, milk, and dairy products [46]. A previous study has reported that in several countries, including Spain, the coastal population generally consumes more fish and seafood products [47]. However, although coastal residents (who have easy access to fish and seafood) consumed slightly more fish and seafood than residents of urban or inland areas, there was no statistically significant difference (*p* = 0.194) between areas.

### 3.2. Adherence to the Atlantic Diet

To evaluate the adherence to the AD, the ADS procedure proposed in this study was applied to data from the FFQ administered to the 456 participants. The results were then compared with the scores obtained using three other assessment methods described in Section 2.5: the SEAD Index by Oliveira et al. [2], the GGP by García-Gómez et al. [8], and the ADS-MBP.

For the ADS procedure (excluding wine), the highest levels of adherence were obtained for the following food categories. First, vegetables, with 74.12% of individuals consuming levels within the adequate range of the OADCVs. Legumes and dairy products were the second group with the highest compliance, with 67.54 and 66.13%, respectively. For meat, 55.26% of respondents met the defined standard. In contrast, the food category that most penalized the final total score was processed food, which only reached 7.46% compliance (since the OADCV for processed food is zero, it should be noted that for this category, it was required not to consume any processed food to obtain a value of 1), and nuts, with 27.63%. Adherence to the recommended range of seafood consumption was 31.36%, which means that just over two-thirds of the population studied ingested less than 50% of the optimal amount of fish in their diet (although in the coastal area, this effect decreases). The price and availability of the product likely affected this finding. Nutritional education actions should be applied in order to increase consumption in this category.

Using the SEAD Index assessment system proposed by Oliveira et al. [2], the level of adherence was highest for consumption of potatoes (77.41% of individuals scored 1), followed by fish (58.77%), meat (47.15%), bread (37.94%), and dairy products (37.28%). These results may appear to differ from those obtained with the ADS, owing to the different composition of the food groups and a very high consumption of cod and vegetable soup by the Portuguese population (both are traditional dishes in Northern Portugal). Moreover, in the ADS, potatoes are included in vegetables, while the SEAD Index includes them as a separate group. Another factor affecting the results is that in the ADS, vegetables and legumes also constitute separate groups, while in the SEAD Index, both foods are included in a single group. This finding indicates the need to standardize the food groups considered, with a clear definition of what is included in each.

Examination of the scores for the 13 items used by the GGP for assessment of AD adherence showed that, as in the ADS, high levels of adherence, of 76.75% and 67.98%, were obtained for vegetables and legumes, respectively, although the highest percentage of adherence was for dairy products (86.84%). Meat (as in the ADS system) is in an intermediate situation (61.84%), while nut consumption was also one of the lowest (39.91%). The categories for which levels of adherence were highest and lowest are similar in both procedures; however, the degree of adherence expressed as a percentage of the total population varies considerably from one system to another, depending on the different criteria used to measure adherence.

Regarding the ADS-MBP, the food groups for which the highest adherence levels were obtained vary in relation to the ADS due to different criteria for qualifying the food partial score as 1 or 0. Specifically, the food categories for which adherence levels were highest were oils/fats and fruit (71.49 and 62.72%, respectively). At the other end of the spectrum, the lowest adherence rate (34.87%) was obtained for seafood. For the remaining categories, adherence ranged from 40% to 60%.

**Figure 4:**
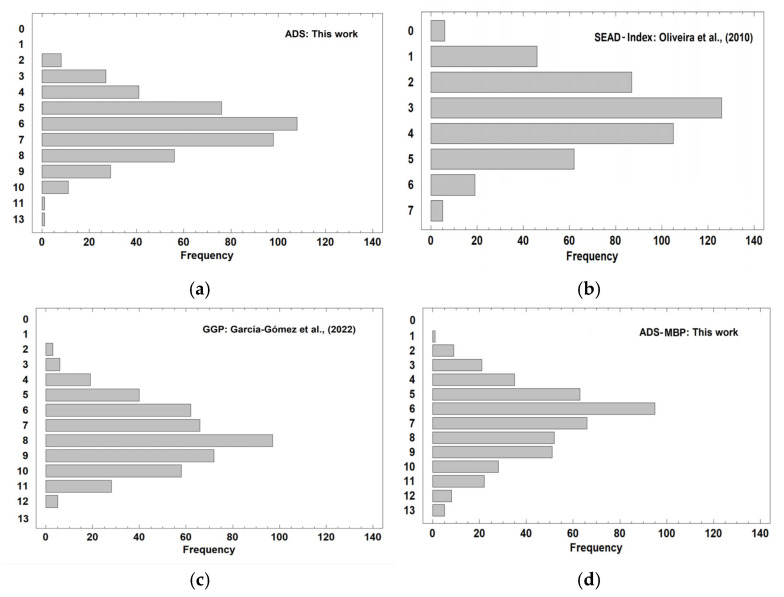
Bar chart showing the indices of adherence to the Atlantic diet for the 456 individuals obtained from (**a**) the ADS method proposed in this paper and (**b**) the SEAD Index procedure developed by Oliveira et al. [2], (**c**) the GGP system by García-Gómez et al. [8], and (**d**) the ADS procedure using the median criteria (ADS-MBP).

The results of the total score adherence indices for the 456 individuals, obtained by the four adherence assessment methods, are presented in Figure 4a–d. The levels of adherence determined in all procedures follow a profile close to normal, with intermediate adherence in most individuals surveyed. In addition, the ADS method, based on a 50–150% range of OADCV standards developed here, yielded similar results to those obtained by using the SEAD Index and ADS-MBP, both based on median values of consumption. These three methodologies also yielded lower levels of adherence than the GGP, which seems to overestimate the degree of adherence.

**Figure 5:**
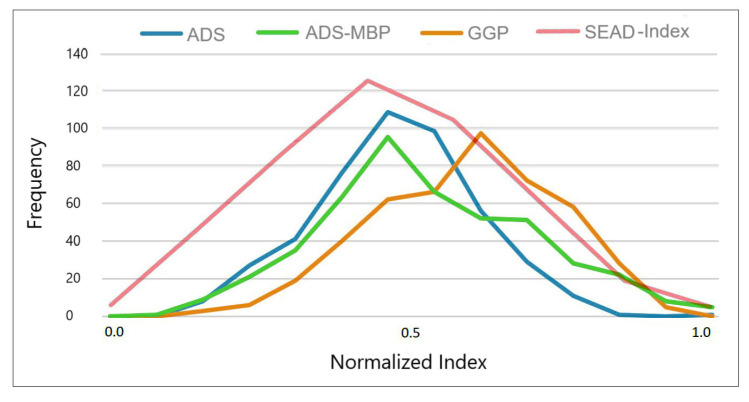
Comparison of normalized indices determined using the different adherence assessment procedures considered.

In order to improve evaluation of the differences among the adherence procedures and given that different scales are used (at least in one of them), a comparison was carried out by means of the normalized index (NI) values whereby the adherence values were normalized according to the scale size between 0 and 1, as indicated above. The results are shown in the plot of the NIs for all adherence systems against the frequency for the 456 individuals tested, shown in Figure 5.

The ADS, SEAD Index, and ADS-MBP yielded similar results, with similar NI profiles. The percentages of individuals with NI higher than 0.5 (which means adherence higher than 50%) for these three adherence assessment procedures were 42.92, 41.88, and 50.87%, respectively. However, the GGP indicated higher adherence levels, and the percentage of cases with NI values greater than 0.5 is 71.49% for this procedure. In contrast, the ADS method proposed here is more demanding than that developed by García-Gómez et al., and in certain cases, even if there is compliance for the corresponding GGP item, the optimal ADS interval is not necessarily reached.

An ANOVA was applied to the normalized index values obtained with the different adherence assessment systems. At least one of the adherence assessment systems produced significantly different (*p* < 0.0001) NI values. The results of the post hoc tests (Tukey’s HSD) indicated that the ADS and the SEAD index produce comparable results, as there is no statistically significant difference between them. The ADS-MBP produced slightly higher values, demonstrating that this procedure generates a higher normalized index than the first two methods. Finally, the GGP stands out for the superior results obtained, significantly outperforming all other methods. The higher NI determined by GGP than by the other three adherence methods can likely be explained by the responses to questions 12 and 13 of the GGP criteria, related to the simple culinary practices and the consumption of local and seasonal products. The gastronomic culture of Galicia and the traditional consumption patterns in this geographical area mostly coincide with these two criteria. As Galician dietary habits are based on simple dishes prepared from local products, high scores were achieved in a great percentage of individuals in these questions. This may have led to overestimation of the final score of the GGP procedure compared to that obtained with the ADS and other methods. Indeed, simulations of the final scores, by eliminating these two very high-compliance items, yielded more similar results (the mean confidence interval (95%) for the ADS was [0.464–0.486], for GGP was [0.580–0.602], and for Corrected-GGP was [0.506–0.527], much closer than the ADS). On the other hand, the comparable results obtained for ADS and the ADS-MBP and SEAD Index indicate that the use of intervals around the OADCV values for characterizing the scores is a better strategy than the use of the sex-specific median value. Thus, in the latter case, the scores must be calculated differently for men and women, while the interval defined in ADS seems to work adequately for both sexes (greatly simplifying the calculation).

### 3.3. Study Limitations

This study had some limitations. The first of these is the sample size considered (456 individuals), which, although large, is smaller than that used in other dietary studies in Galicia (e.g., in [25], where 1601 individuals were surveyed). The gender distribution in our sample, with a greater presence of women than men, does not accurately reflect the demographic composition of the general population. This fact, despite the gender-segregated study conducted in this study, could introduce small biases in the generalizability of the findings. This discrepancy in the male/female ratio is frequently observed in this type of research, where women tend to be more willing to participate in health-related research. Second, the type of survey must be taken into account. The FFQ used in the present study has been widely used to extract dietary information from populations because of the advantages it has over other measurement methods. However, this procedure also has some drawbacks that may bias the results obtained. Due to the period involved in administering the FFQs, there is some dependence on respondents having an accurate memory and ability to accurately estimate the frequency of consumption of foods, especially infrequent ones. FFQs are also influenced by the so-called “social desirability bias”, in which respondents tend to overestimate levels of consumption of healthy foods, such as fruit and vegetables [48]. In addition, FFQs do not usually provide detailed information on the preparation methods of the foods consumed. Third, the procedure for obtaining data and calculating the final score in the ADS procedure is longer and more complex than in the GGP method. Fourth, a limitation of the ADS index is the use of a binary scoring system (0/1) for each food group. While this approach enables straightforward interpretation and facilitates implementation in non-specialized contexts, it may not fully capture small changes in dietary patterns. Future updates to the ADS could include an ordinal scale (points based on intake level) or even a continuous score proportional to the percentage of the recommended intake. These measures could improve the sensitivity and discriminatory capacity of the index at the cost of reducing its practical application. Fifth, it would be important to standardize the definitions of food groups in the different assessment tools to ensure valid comparisons and consistent interpretation of dietary adherence levels. In addition, the different results obtained by the assay methods are due to different considerations and criteria used to define adherence to AD. Therefore, further research using other surveys must be conducted to adequately assess the results.

## 4. Conclusions

The present study achieved its two main objectives. First, in 2022, the current dietary habits of a sample of healthy individuals living in Galicia were determined (GCP-2022). The results were then compared with the optimal Atlantic Diet consumption values (OADCV) established by health authorities. The results revealed improvements in certain food groups (e.g., legumes and nuts) in relation to other previous surveys in Galicia, but overall dietary habits have remained stable. Consumption levels were found to be different from the officially optimum recommended levels (perhaps somewhat demanding) for some food categories, especially regarding fish, grains, and healthy oils. Moreover, the study proposed a new quantitative tool, the Atlantic Diet Scale (ADS), to enable evaluation of adherence to the AD. The ADS method is considered a more flexible alternative than existing approaches since it does not involve strictly defined boundaries but considers that a dietary pattern is met when within an appropriate range. The ADS was applied to the study population, and the results were compared with three other methods of assessing adherence. The adherence levels determined using the ADS were consistently similar to those estimated using the SEAD Index and ADS-MBP, while the method was simpler to use and more accurate. Discrepancies in the final scores were identified relative to the third GGP procedure, influenced by factors considered in this procedure, such as Galician culinary traditions and the consumption of local products, which, in both cases, could contribute to overestimating adherence to AD.

The adherence results produced by the different procedures could hardly be anticipated a priori, due to the different criteria used by each of them. To our knowledge, this is the first study to compare the application of different methods for assessing adherence to the Atlantic diet with the same data. In conclusion, the results of the different assessment methods may vary according to the criteria used. In this regard, the use of ranges around optimal values appears to be a promising approach for a more realistic assessment of dietary adherence, although future research is required to standardize the systems for measuring adherence. Furthermore, longitudinal monitoring of ADS scores over a period of two more years to assess dietary stability could be a very interesting exercise.

Finally, the proposed adherence system (ADS) could be included in public health initiatives in Galicia. Specifically, it could serve as the basis for regionally tailored dietary interventions, including school nutritional programs. Thus, the ADS could be used as a tool to guide meal planning and assess adherence to healthy eating patterns in children and adolescents. Furthermore, the ADS could be used in community workshops and health promotion campaigns as an educational and monitoring tool to promote eating habits aligned with the nutritional guidelines of the Atlantic Diet.

## Figures and Tables

**Table 1:** Optimum Atlantic diet consumption values (OADCV) for the AD provided by the Galician health authorities, and results of the Galician Consumption Pattern survey in the present study (GCP-2022). Statistically significant differences at *p* < 0.01 (**), and *p* < 0.001 (***). ns: not significant.

Food Category	OADCV g/Person/Day [25]	GCP-2022 g/Person/Day This Work	*p*
Fruit	1024	372.94 ± 347.67	***
Vegetables	633	538.64 ± 356.33	***
Legumes	29.3	28.63 ± 38.87	ns
Grains	291	126.94 ± 76.32	***
Nuts	33	20.78 ± 28.86	***
Dairy	419	368.94 ± 227.20	***
Eggs	23.7	30.76 ± 32.94	***
Meat	91.9	107.52 ± 107.20	**
Seafood	195.7	82.98 ± 85.82	***
Processed food	0.00	66.03 ± 85.58	***
Sweets	11.7	47.48 ± 81.51	***
Oil/fats	29.9	15.41 ± 14.48	***

**Table 2:** Items used as criteria in GGP procedure for adherence to AD according to the scheme proposed by García-Gómez et al. [8].

No	Item	Yes	No
1	More than 3 servings/week of fish and/or seafood	1	0
2	More than or equal to 2 servings/day of vegetables and greens	1	0
3	Daily or almost daily potatoes	1	0
4	More than or equal to 3 servings/day of fruit	1	0
5	More than or equal to 2 servings/week of pulses	1	0
6	Daily fresh bread	1	0
7	More than or equal to 4 servings/week of nuts	1	0
8	Daily milk and dairy products	1	0
9	More than 4 servings/week of meat	1	0
10	Olive oil used as the main cooking fat	1	0
11	1 (woman) or 2 (man) glasses of wine/day	1	0
12	Use of simple culinary techniques (boiling, stewing and/or grilling)	1	0
13	Fresh local and seasonal products	1	0

## Data Availability

The raw data supporting the conclusions of this article will be made available by the authors on request.

## Supplementary Materials

The following supporting information can be downloaded at: https://www.mdpi.com/article/10.3390/foods14152614/s1, Table S1: Final sample distribution of the participants according to sex, geographic origin, and age; Table S2. Eigenvalues and cumulative variance explained for the principal component analysis. Table S3. Statistical data for consumption of the different food categories in relation to sex, age and location.
