# Supplementary material for: Observance of the Atlantic Diet in a Healthy Population from Galicia (NW Spain): A Comparative Study Using a New Scale-Based Procedure to Assess Adherence
Source: Foods. 2025 Jul 25;14(15):2614. doi: 10.3390/foods14152614 (PMC12346738; doi:10.3390/foods14152614)
Supplement: Supplementary file 1 [file foods-14-02614-s001.zip › foods-3702135-supplementary.pdf]

Observance to the Atlantic diet in healthy population of Galicia (NW Spain): a comparison study using a new range-based adherence assessment procedure

Inés Rivas-Fernández[1], Paula Roade-Pérez[2], Marta López-Alonso[3], Víctor Pereira-Lestayo[3], Rafael Monte-Secades[1], Rosa Argüeso-Armesto[1], Carlos Herrero-Latorre[4]

[1]1 Hospital Universitario Lucus Augusti. Servizo Galego de Saúde. Xunta de Galicia. Calle Dr. Ulises Romero 1. 27003 Lugo, Spain.
2 Departamento de Química Analítica, Nutrición y Bromatología. Facultade de Veterinaria, Campus Terra, Universidade de Santiago de Compostela. 27002 Lugo, Spain.
3 Departamento de Patoloxía Animal, Facultade de Veterinaria, Campus Terra, Universidade de Santiago de Compostela. 27002 Lugo, Spain
4 Aquatic One Health Research Center (ARCUS), Departamento de Química Analítica, Nutrición y Bromatología, Facultade de Ciencias, Campus Terra, Universidade de Santiago de Compostela. 27002 Lugo, Spain.
* Correspondence: victor.pereira@usc.es

SUPPLEMENTARY MATERIAL

Table S1. Final sample distribution of the participants according to sex, geographic origin, and age.

**Sex**	**n**	**Geographic origin**	**n**	**Age**	**n**
Female	295	Coastal	81	18-39	123
Male	161	Inland	90	40-59	241
		Urban	285	≥60	92
TOTAL	456		456		456

Table S2. Eigenvalues and cumulative variance explained for the principal component analysis.

Principal Component	Eigenvalue	Variance (%)	Cumulative Variance (%)
1	2.4318	18.706	18.706
2	1.9523	15.018	33.724
3	1.2482	9.602	43.326
4	1.1803	9.080	52.406
5	1.0168	7.822	60.227
6	0.9357	7.198	67.426
7	0.8642	6.648	74.074
8	0.8250	6.347	80.421
9	0.7627	5.867	86.288
10	0.6362	4.894	91.182
11	0.5833	4.488	95.670
12	0.5322	4.094	99.764
13	0.0306	0.236	100.000

Table S3. Statistical data for consumption of the different food categories in relation to sex, age and location.

Category	Sex	Media	CV (%)	Geog. Area	Media	CV (%)	Age	Media	CV (%)
**Fruits**	Female	399.1	83.56	Coastal	408.5	75.7	18-39	348.7	96.35
	Male	357.3	102.7	Inland	401	86.83	40-59	372.93	93.18
				Urban	372.3	95.78	≥60	462	75.84
**Vegetables**	Female	586.4	62.84	Coastal	527.3	58.76	18-39	55.6	59.7
	Male	439.2	60.7	Inland	593.2	59.45	40-59	545.7	58.54
				Urban	551	64.97	≥60	585.8	74.99
**Legumes**	Female	28.17	109.3	Coastal	27.57	113	18-39	27.4	123.1
	Male	31.96	159.6	Inland	34.54	150.8	40-59	28.71	121.9
				Urban	28.47	127.5	≥60	34.4	155.6
**Grains**	Female	129.2	57.03	Coastal	111.8	69.59	18-39	146.7	53.01
	Male	133.7	56.19	Inland	144.6	53.59	40-59	130.1	56.14
				Urban	131.8	53.85	≥60	111.3	60.82
**Nuts**	Female	22.55	132.4	Coastal	18.78	141.1	18-39	21.5	159
	Male	19.32	142.3	Inland	21.61	110.2	40-59	20.79	130.2
				Urban	22.09	141.3	≥60	22.89	117
**Dairy**	Female	380.1	58.8	Coastal	329.5	68.78	18-39	407.6	49.31
	Male	38.055	56.9	Inland	419.4	53.11	40-59	375.6	61.99
				Urban	382.3	56.75	≥60	355.7	59.99
**Eggs**	Female	29.7	80.5	Coastal	28.12	127.9	18-39	40.28	105.6
	Male	35.34	127.2	Inland	31.17	105.8	40-59	31.25	80.51
				Urban	36.57	96.13	≥60	24.01	149.3
**Meat**	Female	100.7	79.27	Coastal	96.48	78.35	18-39	121.8	81.59
	Male	120.2	142.7	Inland	145.4	119.2	40-59	98.82	69.73
				Urban	103.9	84.64	≥60	127.5	137.6

Category	Sex	Media	CV (%)	Geog. Area	Media	CV (%)	Age	Media	CV (%)
**Seafood**	Female	87.55	92.65	Coastal	99.19	79.92	18-39	74.38	80.22
	Male	81.79	115	Inland	89.57	113.2	40-59	84.86	93.04
				Urban	80.35	102.1	≥60	102.1	120.7
**Proc food**	Female	71.17	132.9	Coastal	54.35	107.8	18-39	80.11	82.17
	Male	63.95	108.2	Inland	69.73	194.2	40-59	66.33	148
				Urban	72.33	99.69	≥60	59.28	129.5
**Sweets**	Female	52.96	173.8	Coastal	38.79	142.3	18-39	50.41	120
	Male	43.03	144	Inland	50.22	259.2	40-59	49.08	194.7
				Urban	52.24	132.1	≥60	49.16	147.5
**Oil/fats**	Female	17.84	83.92	Coastal	15.29	86.32	18-39	17.12	94.47
	Male	12.27	103.2	Inland	15.46	110.7	40-59	15.86	85.43
				Urban	16.17	85.86	≥60	14.26	99.91
**Wine**	Female	29.95	260.6	Coastal	36.24	255.3	18-39	15.64	300.5
	Male	89.81	202.9	Inland	69.95	246.3	40-59	37.32	220.7
				Urban	49.39	243.5	≥60	134.5	169.7
